# Rapamycin as a Potential Alternative Drug for Squamous Cell Gingiva Carcinoma (Ca9-22): A Focus on Cell Cycle, Apoptosis and Autophagy Genetic Profile

**DOI:** 10.3390/ph17010131

**Published:** 2024-01-19

**Authors:** Sofia Papadakos, Hawraa Issa, Abdulaziz Alamri, Abdullah Alamri, Abdelhabib Semlali

**Affiliations:** 1 Groupe de Recherche en Écologie Buccale, Faculté de Médecine Dentaire, Université Laval, Québec, QC G1V 0A6, Canada; sofia.papadakos.1@ulaval.ca (S.P.); hawraa.issa.1@ulaval.ca (H.I.); 2 Biochemistry Department, College of Science, King Saud University, P.O. Box 2455, Riyadh 11451, Saudi Arabia; abalamri@ksu.edu.sa (A.A.); abdullah@ksu.edu.sa (A.A.)

**Keywords:** Rapamycin, oral cancer, cell cycle, apoptosis, autophagy, genetic profile

## Abstract

Oral cancer is considered as one of the most common malignancies worldwide. Its conventional treatment primarily involves surgery with or without postoperative adjuvant therapy. The targeting of signaling pathways implicated in tumorigenesis is becoming increasingly prevalent in the development of new anticancer drug candidates. Based on our recently published data, Rapamycin, an inhibitor of the mTOR pathway, exhibits selective antitumor activity in oral cancer by inhibiting cell proliferation and inducing cancer cell apoptosis, autophagy, and cellular stress. In the present study, our focus is on elucidating the genetic determinants of Rapamycin’s action and the interaction networks accountable for tumorigenesis suppression. To achieve this, gingival carcinoma cell lines (Ca9-22) were exposed to Rapamycin at IC_50_ (10 µM) for 24 h. Subsequently, we investigated the genetic profiles related to the cell cycle, apoptosis, and autophagy, as well as gene–gene interactions, using QPCR arrays and the Gene MANIA website. Overall, our results showed that Rapamycin at 10 µM significantly inhibits the growth of Ca9-22 cells after 24 h of treatment by around 50% by suppression of key modulators in the G2/M transition, namely, Survivin and CDK5RAP1. The combination of Rapamycin with Cisplatin potentializes the inhibition of Ca9-22 cell proliferation. A P1/Annexin-V assay was performed to evaluate the effect of Rapamycin on cell apoptosis. The results obtained confirm our previous findings in which Rapamycin at 10 μM induces a strong apoptosis of Ca9-22 cells. The live cells decreased, and the late apoptotic cells increased when the cells were treated by Rapamycin. To identify the genes responsible for cell apoptosis induced by Rapamycin, we performed the RT^2^ Profiler PCR Arrays for 84 apoptotic genes. The blocked cells were believed to be directed towards cell death, confirmed by the downregulation of apoptosis inhibitors involved in both the extrinsic and intrinsic pathways, including BIRC5, BNIP3, CD40LG, DAPK1, LTA, TNFRSF21 and TP73. The observed effects of Rapamycin on tumor suppression are likely to involve the autophagy process, evidenced by the inhibition of autophagy modulators (TGFβ1, RGS19 and AKT1), autophagosome biogenesis components (AMBRA1, ATG9B and TMEM74) and autophagy byproducts (APP). Identifying gene–gene interaction (GGI) networks provided a comprehensive view of the drug’s mechanism and connected the studied tumorigenesis processes to potential functional interactions of various kinds (physical interaction, co-expression, genetic interactions etc.). In conclusion, Rapamycin shows promise as a clinical agent for managing Ca9-22 gingiva carcinoma cells.

## 1. Introduction

Oral cancer (OC) ranks among the most common malignancies, primarily attributed to tobacco use, alcohol consumption and the consumption of areca nut (betel quid). Annually, 380,000 new cases are diagnosed globally [1], with the majority concentrated in Asia at 64.2%, trailed by Europe at 17.4% and North America at 7.6% [2]. The choice of care strategy depends on factors such as the disease stage, tumor size, patient age and health, and potential outcomes in terms of appearance and functional abilities [3]. For early stages (I and II), standard treatment involves surgery or radiation therapy, while a combination of both is preferred for advanced stages (stages III and IV) [3]. Surgical interventions may impact functional tasks such as eating, drinking and talking [4]. In cases of recurrence or distant metastases, chemotherapy drugs can be added to the previously denoted plan [3] in the hope of extending life expectancy [4]. One of the best and most widely used chemotherapeutic drugs is Cisplatin, a molecule that exerts its anticancer effect by causing DNA damage. Although it is effective, patients receiving Cisplatin may experience resistance to the drug, along with a range of side effects, including serious kidney complications, allergic reactions, compromised immune system and intestinal issues [5], anemia, appetite loss and fertility issues. Although chemotherapy is often associated with better overall survival rates in patients with OC, its clinical use is limited by its severe and toxic dose-dependent side effects. Owing to the unavailability of effective treatments that are free of side effects, OC develops a strong tendency toward drug resistance and metastases. Thus, finding new anticancer drugs for the treatment of OC is considered an emergency approach.

Recent works proposed targeting deregulated cancer pathways. In particular, the PI3K/AKT/mTOR (phosphoinositide 3-kinase/protein kinase B/ mammalian (or mechanistic) target of Rapamycin) was found to be hyperactivated in almost all malignant neoplasms [6] with a prevalence ranging from 40% to 90%, thus rendering it an appealing target for cancer treatment [7]. Hence, many studies have designed anticancer agents targeting elements of the PI3K/AKT/mTOR pathway [6]. A certain number of these inhibitors have been studied in several human cancer models and have successfully led to tumor regression [8]. This is achievable though the control of various cellular functions, including but not limited to protein synthesis, cell division, autophagy, programmed cell death, migration, invasion and angiogenesis [9]. While the stimulation of both complexes of mTOR, mTORC1 and mTORC2, generally promotes cell growth and viability [10], inhibiting mTOR was proven to help reestablish cellular balance and to support antigen presentation and formation of memory T cell populations [7].

On this account, the mTOR inhibitor Rapamycin has shown promise in managing numerous tumors and hematologic malignancies [6]. This molecule, first isolated in the late 1960s from soil samples, originates from the bacteria *Streptomyces hygroscopicus* [11]. Initially approved by the Food and Drug Administration (FDA) for its immunosuppressive properties, Rapamycin has been used to prevent kidney transplant rejection, treat pulmonary lymphangioleiomyomatosis and address tuberous sclerosis-associated fibrous skin tumors [12]. Subsequent research revealed that this drug slows or stops the growth of several cancers affecting skeletal muscles, bones, pancreas, breasts, prostates and B-lymphocytes [13]. Hence, the FDA granted approval in 2011 for its use in managing pancreatic cancer [11]. Rapamycin’s anticancer potential emerges from its capacity to selectively target mTORC1 [9]. In a previous study, our team showcased its inhibitory effects on cell growth, formation of colonies, cellular adhesion, inflammatory responses and cellular migration, while promoting cell death and autophagy in oral cancer cells [14]. On this basis, the primary objective of the present study was to explore the genetic determinants of Rapamycin’s action on Ca9-22 gingival squamous carcinoma cells in vitro. For this purpose, key autophagy, apoptosis and cell cycle modulators were explored and the possible genetic interaction networks were established. Moreover, the potential beneficial impact of a Rapamycin–Cisplatin combination treatment was examined.

## 2. Results

### 2.1. Rapamycin Exhibits Anti-Oral Cancer Activity through Stimulation of Apoptosis and Autophagy

**Figure 1:**
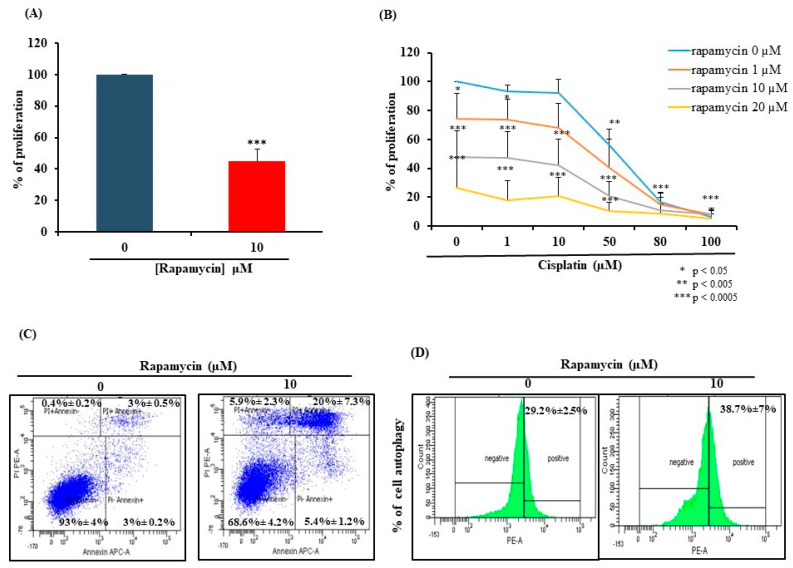
Effect of Rapamycin on Ca9-22 cell viability, apoptosis, and autophagy. (**A**) Effect of Rapamycin on cell growth evaluated by MTT cell proliferation and viability assay. The presented data are expressed as mean ± SEM values of 6 independent experiments. *** *p* < 0.001 is considered statistically significant. (**B**) Synergistic effect of Rapamycin and Cisplatin combinations on cell growth revealed by MTT assay (*n* = 4). (**C**) Apoptosis assessment conducted using the PI and Annexin markers. Results are expressed as mean ± SEM of 4 independent experiments. (**D**) Flow cytometry analysis showing the percentage of autophagic cells. Results are expressed as mean ± SEM of 4 independent experiments.

Based on our published data [14], Rapamycin’s IC_50_ was recorded at 10 µM. As shown in Figure 1A, the supplementation of Rapamycin at this specific concentration significantly inhibited the growth of Ca9-22 cells by approximately 50%. Notably, the combination of Rapamycin with Cisplatin potentializes Cisplatin’s effect on gingiva carcinoma cell proliferation as per the MTT test results (Figure 1B).

This enhancement can be attributed to Rapamycin’s ability to induce cell death through apoptosis after a 24 h treatment. At this level, a notable rise in the number of cells in the late apoptotic stage was observed, rising from 3% ± 0.5% to 20% ± 7.3% (Figure 1C). Moreover, Rapamycin at 10 µM exhibited a notable effect on cell autophagy, with the percentage of autophagic cells increasing from 29.2% ± 2.5% to 38.7% ± 7% upon stimulation with Rapamycin (Figure 1D).

### 2.2. Rapamycin’s Action Is Mediated through the Repression of Cell Division Modulators BIRC5 and CDK5RAP1

**Figure 2:**
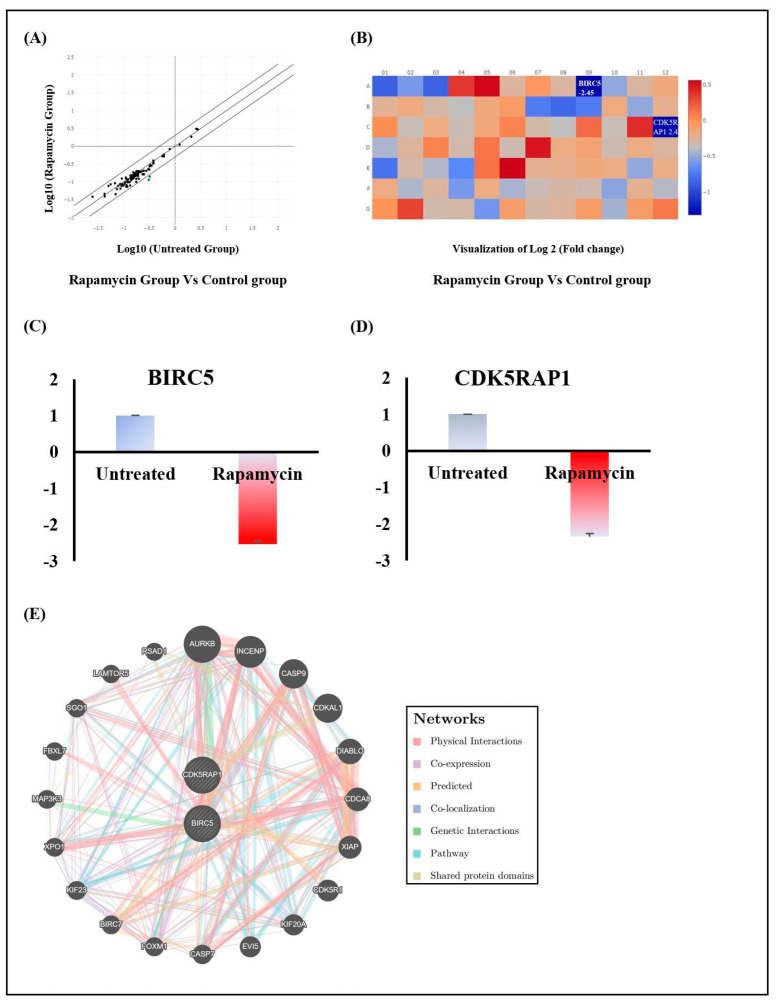
Effect of Rapamycin on Ca9-22 cell cycle arrest markers. (**A**) Differences in the expression of cell cycle-related genes. QPCR array screening for 84 markers after incubation with 10 µM Rapamycin (*n* = 3). (**B**–**D**) Summary of positively and negatively modulated genes. Only fold regulation values above 2 were considered (*n* = 3). (**E**) Interaction networks of Rapamycin-regulated genes involved in the cell cycle.

To investigate the potential effect of Rapamycin on cell cycle arrest, a screening of 84 genes involved in this process was realized using the QIAGEN cell cycle RT 2 Profiler PCR Arrays. At least two-fold differential gene expression between Rapamycin-treated cells and untreated cells was taken into consideration in our analysis (Figure 2A). Overall, only two genes, recognized as cell cycle promoters and apoptosis inhibitors, were modulated by the action of Rapamycin (Figure 2B). Specifically, the Baculoviral inhibitor of apoptosis repeat-containing 5 (BIRC5) and the CDK5 regulatory subunit-associated protein (CDK5RAP1) were inhibited, with changes of −2.53-fold ±0.08 and −2.34-fold ±0.05, respectively (Figure 2C,D). A detailed analysis of the interaction networks revealed that BIRC5 and CDK5RAP1 are closely connected to 20 other genes. This includes aurora kinase B (AURKB), inner centromere protein (INCENP), caspase 9 (CASP9), CDK5 regulatory subunit associated protein 1 like 1 (CDKAL1), diablo IAP-binding mitochondrial protein (DIABLO), cell division cycle associated 8 (CDCA8), X-linked inhibitor of apoptosis (XIAP), cyclin dependent kinase 5 regulatory subunit 1 (CDK5R1), kinesin family member 20A (KIF20A), ectotrophic viral integration site 5 (EVI5), caspase 7 (CASP7), forkhead box M1 (FOXM1), baculoviral IAP repeat containing 7 (BIRC7), kinesin family member 23 (KIF23), exportin 1 (XPO1), mitogen-activated protein kinase 3 (MAP3K3), F-box and leucine rich repeat protein 7 (FBXL7), shugoshin 1 (SGO1), late endosomal/lysosomal adaptor, MAPK and MTOR activator 5 (LAMTOR5) and radical S-adenosyl methionine domain containing 1 (RSAD1). In terms of interaction nature, seven types of GGI were observed between the various components of this network, including physical interaction, co-expression, predicted, co-localization, genetic interactions, pathway and shared protein domain (Figure 2E).

### 2.3. Rapamycin Induces Gingiva Carcinoma Cell Apoptosis via Downregulation of Intrinsic and Extrinsic Apoptotic Pathways Components

**Figure 3:**
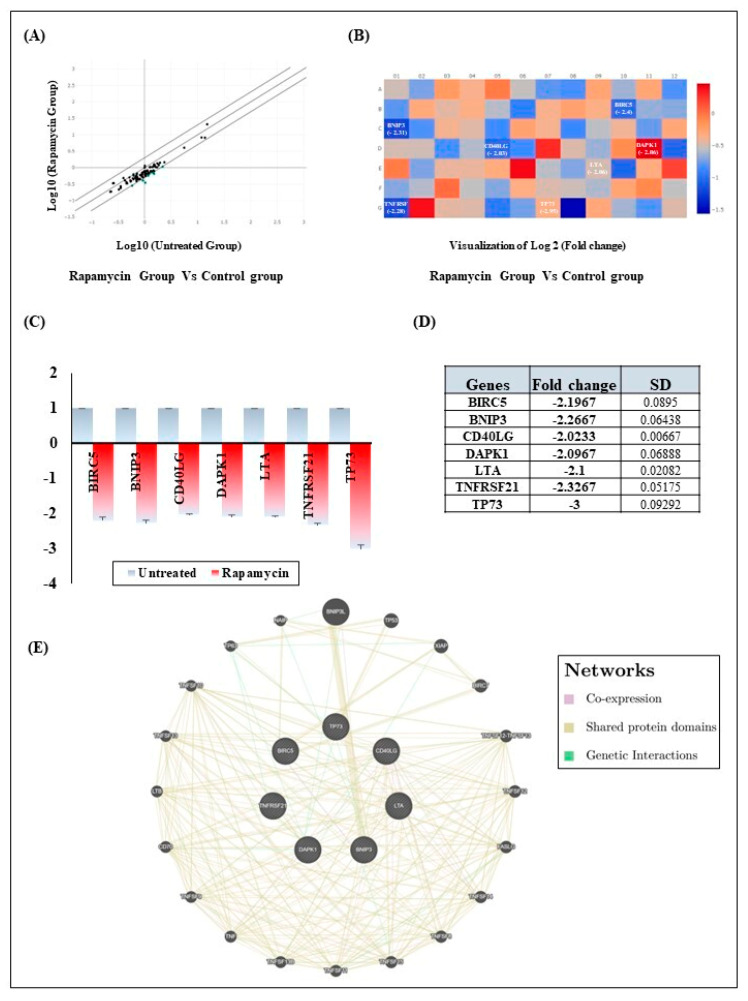
Effect of Rapamycin on Ca9-22 apoptosis markers. (**A**) Variations in the apoptosis-related gene expression was detected in Ca9-22 cells, both in untreated conditions and those treated with Rapamycin, as determined by QPCR array. A total of 84 markers were included in the screening (*n* = 3). (**B**,**C**) Visualization of log2 fold changes following exposure to Rapamycin relative to untreated cells (*n* = 3). (**D**) Table showing the list of genes modulated by Rapamycin as well as the associated fold change (*n* = 3). (**E**) Network of gene interactions influenced by Rapamycin, specifically those associated with apoptosis.

To investigate the determinants of the pro-apoptotic effect of Rapamycin in Ca9-22 cells, the RT^2^ Profiler PCR Arrays were employed. Upon screening for 84 apoptotic genes, seven markers were shown to be downregulated at least two-fold (Figure 3A,B), namely, BIRC5 (−2.2-fold), Bcl-2 interacting protein 3 (BNIP3) (−2.26-fold), CD40 Ligand (CD40LG) (−2.02-fold), death-associated protein kinase 1 (DAPK1) (−2.09-fold), lymphotoxin alpha (LTA) (−2.1-fold), tumor necrosis factor receptor superfamily member 21 (TNFRSF21) (−2.32-fold) and tumor protein 73 (TP73) (3-fold) (Figure 3C,D). These factors are known components of both the extrinsic and intrinsic pathways and are believed to form interaction networks with 20 other genes involved in apoptosis. This includes BCL2 interacting protein 3 like (BNIP3L), tumor protein p53 (TP53), X-linked inhibitor of apoptosis (XIAP), baculoviral IAP repeat containing 7 (BIRC7), tumor necrosis factor (TNF), TNF superfamily member 8 (TNFSF8), TNF superfamily member 8 (TNFSF9), TNF superfamily member 8 (TNFSF10), TNF superfamily member 11 (TNFSF11), TNF superfamily member 12 (TNFSF12), TNFSF12-TNFSF13 readthrough (TNFSF12-TNFSF13), TNF superfamily member 13 (TNFSF13), TNF superfamily member 13b (TNFSF13B), TNF superfamily member 14 (TNFSF14), TNF superfamily member 15 (TNFSF15), Fas ligand (FASLG), CD70 molecule (CD70), lymphotoxin beta (LTB), tumor protein p63 (TP63) and NLR family apoptosis inhibitory protein (NAIP). The reported interactions are of three types: (i) co-expression, as in the case of BNIP3-BNIP3L, or CD40LG with either the LTA gene or the TNF family members TNFSF11 and TNFSF15; (ii) shared protein domains, illustrated in the following examples: BNIP3-BNIP3L or DAPK1 sharing protein domains with the TNF family; and (iii) genetic interactions, observed between BNIP and either NAIP or TP73 or between DAPK1 and either TP63 or BNIP3L (Figure 3E).

### 2.4. Rapamycin Treatment Induces Gingiva Carcinoma Cell Autophagy by Inhibition of Seven Autophagy Genes

**Figure 4:**
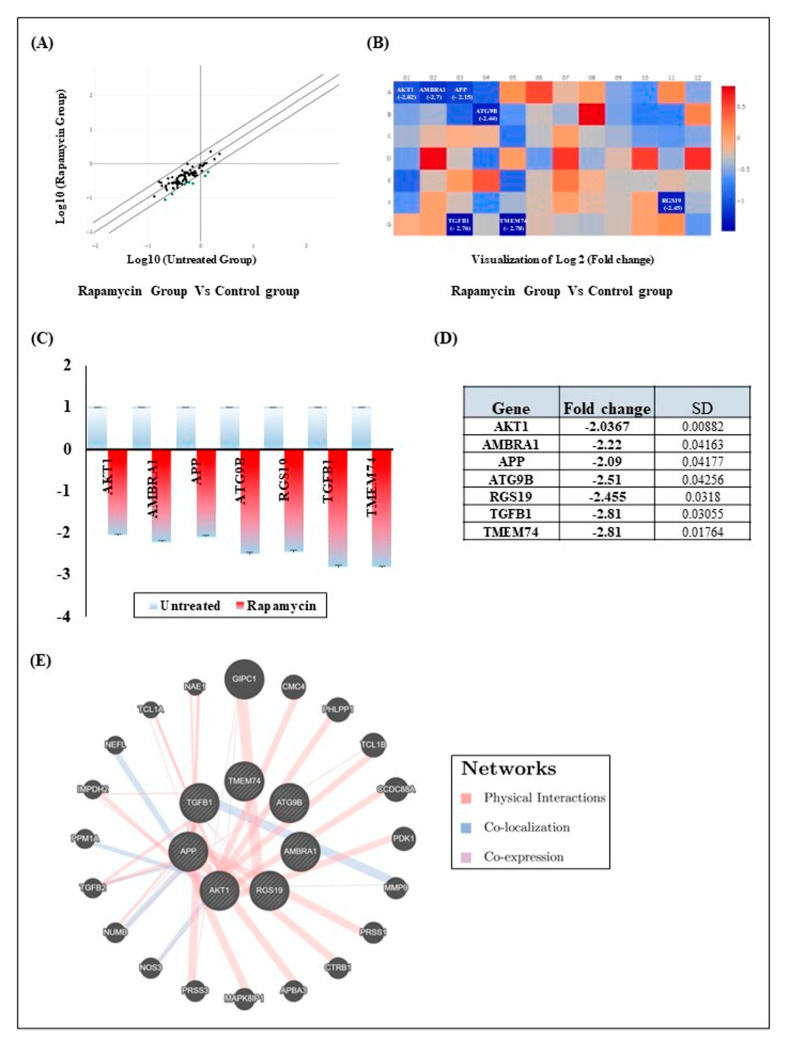
Effect of Rapamycin on Ca9-22 autophagy markers. (**A**) Discrepancies in the expression genes related to autophagy were investigated in Ca9-22 cells under untreated conditions and after treatment with Rapamycin. QPCR array screening for 84 markers was conducted following incubation with 10 µM of Rapamycin (*n* = 3). (**B**,**C**) Visualization of significant changes induced by Rapamycin as compared to untreated cells (*n* = 3). (**D**) Display of the genes downregulated by Rapamycin along with the change magnitude (*n* = 3). (**E**) Graphical representation of the autophagy gene interaction networks, particularly those linked to the modulated markers.

A transcriptomic study using QPCR arrays to examine factors involved in Rapamycin-modulated autophagy revealed the downregulation of seven genes (Figure 4A,B). Specifically, two of these genes, autophagy/beclin 1 regulator 1 (AMBRA1) (−2.22-fold) and autophagy related 9B (ATG9B) (−2.51-fold), were identified as autophagy machinery components. The remaining five genes are key regulators of both autophagy and apoptosis: AKT serine/threonine kinase 1 (AKT1) (−2.03-fold), amyloid-beta precursor protein (APP) (−2.09-fold), regulator of G protein signaling 19 (RGS19) (−2.45-fold), transforming growth factor-beta 1 (TGFB1) (−2.81-fold) and transmembrane protein 74 (TMEM74) (−2.81-fold) (Figure 4C,D). These factors interact closely with a total of 20 other genes involved in cell apoptosis and autophagy, including GAIP-interacting protein C-terminus 1 (GIPC1), C-X9-C motif containing 4 (CMC4), PH domain and leucine rich repeat protein phosphatase 1 (PHLPP1), T-cell leukemia/lymphoma protein 1B (TCL1B), coiled-coil domain containing 88A (CCDC88A), pyruvate dehydrogenase kinase 1 (PDK1), matric metallopeptidase 9 (MMP9), protease serin 1 (PRSS1), chymotrypsinogen B1 (CTRB1), amyloid beta precursor protein binding family A member 3 (APBA3), mitogen-activated protein kinase 8 interacting protein 1 (MAPK8IP1), serine protease 3 (PRSS3), nitric oxide synthase (NOS3), NUMB endocytic adaptor protein (NUMB), transforming growth factor beta 2 (TGFB2), protein phosphatase 1A (PPM1A), inosine monophosphate dehydrogenase 2 (IMPDH2), neurofilament light chain (NEFL), T-cell leukemia/lymphoma protein 1A (TCL1A) and NEDD8 activating enzyme E1 subunit 1 (NAE1). At this level, three modes of network interaction were observed: (i) physical interactions between GIPC1-RGS19 or AKT1-CCDC88A; (ii) co-localization of AKT1 and NOS3 or PPM1A; and (iii) co-expression of AKT1-GIPC1 or TGFB1-RSG19 (Figure 4E).

## 3. Discussion

Anticancer strategies targeting tumorigenesis signaling pathways have garnered significant interest within the scientific community. The PI3K/AKT/mTOR, in particular, has drawn attention due to its involvement in the pathogenesis of various malignancies. Having gained approval for pancreatic cancer treatment, we have suggested that the mTOR inhibitor Rapamycin could be adopted as a potential treatment regimen for oral cancer patients due to its promising effects. In fact, our recent study demonstrated that this drug inhibits the proliferation of squamous cell gingiva carcinoma while inducing apoptosis and autophagy in vitro [14]. Investigating the genetic background in play is crucial to understanding the mechanistic basis for eventual drug approval. As far as we are aware, this study is the first to investigate the transcriptomic profile of Rapamycin-modulated genes, particularly those governing cell cycle progression, apoptosis, and autophagy.

With regards to Rapamycin’s impact on cell proliferation, our investigation raised questions about its potential influence on cell cycle modulators. The QPCR array results revealed the downregulation of BIRC5 and CDK5RAP1. Given that BIRC5 increases at the centromeres level during G2/M transition and that it is believed to perform its mitotic role through modulation of microtubule function and participation in spindle checkpoint regulation [15] and taking into account that a CDK5RAP1 deficiency suppresses tumor growth through G2/M cell cycle arrest [16], we propose that the treatment with Rapamycin promotes G2/M-phase arrest of oral cancer cells by suppressing BIRC5 and CDK5RAP1. An established fact is that it is only normal to direct the arrested cell to death. On one hand, it is documented that repression of CDK5RAP1, an inhibitor of cyclin-dependent kinase 5 activity, forces malignant melanoma and human breast cancer cell lines to undergo apoptosis [16,17]. The same applies to the inhibitors of apoptosis (IAP) family member BIRC5 [18]. In this regard, decreasing BIRC5′s oncogene expression might correct the resistance to various therapy regimens [19] and serve as a promising independent cancer survival marker [20]. On this basis, several selective inhibitors of BIRC5 (a regulator of autophagy) have been generalized to manage cancer by disrupting BIRC5′s homodimerization or interaction with partner proteins [21]. The latter can be any of the 20 genes revealed by studying the GGI networks. In fact, complex interactions of BIRC5 and CDK5RAP1with members of the IAP family and cell cycle modulators among others might play an important role in cell division control or apoptosis induction. For instance, antagonizing IAP proteins with Diablo promotes caspase activation, thus facilitating programmed cell death [22]. This involves caspase-9, an initiator caspase implicated in apoptosis signal transduction [23]. Another IAP family member that is highly likely to take part is XIAP [24]. As for the cell division regulators, examples include the chromosomal passenger complex components CDCA8, AURKA and INCENP. These oncogenes are correlated with aggressive features and are essential for cancer cell survival, proliferation, metastasis and apoptosis [25,26,27].

Based on the above, it was important to scan for a larger group of genes implicated in apoptosis. As per the QPCR array data, Rapamycin is believed to induce apoptosis in cancer cells by altering the components of the extrinsic (CD40LG, DAPK1, LTA, TNFRSF21) and intrinsic (BIRC5, BNIP3) apoptotic pathways as well as the TP73 upstream modulator. This TP73 transcription factor shares structural and functional similarities with the tumor suppressor p53 and is known to control multiple processes including apoptosis, cell cycle arrest and DNA repair. Its decreased expression due to Rapamycin’s action is expected to hinder metastasis and invasion [28]. For the intrinsic mitochondrial pathway, it is anticipated that the downregulation of BIRC5 will direct the cells towards apoptosis after blocking G2/M transition [29] while BNIP3 suppression may be associated with less aggressive tumors [30]. This Bcl-2 protein family member is upregulated in many types of cancer [31,32,33,34] and is implicated in different cell death pathways, including apoptosis, necrosis and autophagy [35]. Regarding the extrinsic pathway, TNF superfamily ligands (CD40LG, LTA) and receptor TNFRSF21 as well as their downstream effector DAPK1 were shown to be downregulated. Although CD40LG and LTA exhibit dichotomous context-dependent actions on tumor progression [36,37], the efficiency of Rapamycin at this level may emerge from its immunomodulatory function. Of note, both ligands are known to play a key role in chronic inflammation where aberrant expression contributes to tumor growth and immune evasion [36,38,39,40]. A dualistic role in terms of apoptosis induction also applies for TNFRSF21 depending on the specific cellular and microenvironmental conditions, as well as the interplay with signaling pathways [41]. This receptor is reported to act as a promoter for cell survival and a contributor to resistance against apoptosis-inducing anticancer therapies [42]. As for DAPDK1 downregulation by Rapamycin despite its identification as a tumor suppressor, a plausible explanation might be linked to its pro-survival activity [43]. At this level, some studies proposed a positive effect of DAPK1 on mTORC1 signaling in response to growth factors [44]. Added complexity can emerge from the possible interactions with other apoptotic genes such as BNIP3L, TP53, XIAP, BIRC7 and TNFSF12 among others. The functions are so diverse and can cover mitophagy, cellular homeostasis, cell cycle progression, DNA repair and apoptosis [45,46,47,48]

Autophagy being an essential catabolic process controlled by Rapamycin [14], it was highly important to identify the genes responsible for cellular recycling and removal of damaged organelles and proteins. Overall, autophagy modulators (TGFβ1, RGS19 and AKT1), autophagosome biogenesis components (AMBRA1, ATG9B and TMEM74) and autophagy byproducts (APP) were confirmed to be downregulated by QPCR arrays. Although it was demonstrated that TGFβ contributes to tumor suppression through induction of autophagy [49], reports also suggests an inhibitory feedback loop reducing TGFβ expression [50]. While its tumor-suppressive role is often observed in the early stages of cancer development, the inhibitory effects of Rapamycin may stem from TGFβ’s capacity to fuel tumor progression in later stages, by promoting angiogenesis, immune evasion and metastasis [51,52]. At this level, inhibition of Akt phosphorylation blocks cell surface translocation of TGFβ receptors residing in the cytosol thus reducing responsiveness to TGFβ. Based on the fact that TGFβ receptors present the possibility of being transactivated by G protein-coupled receptors (GCPR) [53], this might explain the observed repression of the RGS19 component of GPCR signaling [54,55]. On the other hand, suppressing AKT1 by Rapamycin is believed to activate autophagosome formation by stimulating the ULK1 complex [56].

Other critical genes implicated in autophagosome formation are AMBRA1, ATG9B and TMEM74. While TMEM74, an autophagosome- and lysosome-associated transmembrane protein, induces cell autophagy, with the detailed mechanism remaining unclear [57], AMBRA1 interacts with the Beclin 1-VPS34 complex and promotes autophagosome formation. This multifunctional protein is not just implicated in triggering autophagy; it also contributes to the regulation of autophagic flux and ensures the proper execution of the autophagic process [58]. On the other hand, orchestrated by signaling pathways such as ULK1 [59], ATG9B shuttling between the trans-Golgi network and endosomes membranes is crucial for autophagosome formation. ATG9B dynamics and transient interactions with autophagosomes are vital for the efficient execution of autophagy, ensuring proper cargo encapsulation and autophagosome maturation [60]. Although AMBRA1, ATG9B and TMEM74 suppression by Rapamycin signals defects in autophagy, evidence suggests the existence of a self-regulatory loop directing tumor cells to death rather than autophagy [61,62,63]. Given that functional autophagy is linked to clearance of the APP precursor of toxic protein aggregates [64] and that APP overexpression was associated with lower survival rates in various cancer types due to its involvement in cancer proliferation and invasion [65,66], its downregulation by Rapamycin was awaited.

Investigations of the autophagy gene interaction networks suggest possible connections with metastasis, angiogenesis and inflammation control. For instance, the TCLB1 gene regulates T-cell biology, influencing proliferation and survival [67], while CCDC88A modulates Wnt signaling and promotes epithelial–mesenchymal transition, conferring a metastasis potential [68]. This can be aggravated by the expression of matrix metalloproteinases, such as MMP9, known to facilitate extracellular matrix degradation and to promote invasion as well as angiogenesis [69,70].

## 4. Materials and Methods

### 4.1. Cell Culture

The gingival epithelial squamous carcinoma cell line (Ca9-22) was purchased from the RIKEN BioResource Research Center (Tsukuba, Japan). These cells were cultured in RPMI-164 medium (Gibco, Waltham, MA USA) supplemented with 5% fetal bovine serum (Gibco, 12483-020), 1% penicillin–streptomycin (Sigma, Oakville, ON, Canada) and 1% Fungizone (Sigma, A2942). The Ca9-22 cells were kept at 37 °C in a controlled humidity environment containing 5% CO_2_ and the culture medium was refreshed every other day until reaching 90% of confluence. Rapamycin (MedChemExpress, Monmouth Junction, NJ, USA) and Cisplatin (MedChemExpress, HY-17394) were incubated with the cells for 24 h.

### 4.2. Cell Viability Assay

Cell growth was evaluated using the MTT assay, as outlined in prior works [14,71,72,73]. Briefly, the Ca9-22 cells were exposed to Rapamycin, whether alone or in combination with different concentrations of Cisplatin (1 to 100 µM), for 24 h. The medium was exchanged with a fresh solution containing 0.5 mg/mL 3-(4,5-dimethylthiazol-2-yl)-2,5-diphenyltetrazolium bromide MTT agent (Sigma, M-2128) before incubating the cells at 37 °C. Following three hours in the dark, the purple formazan product generated by metabolically active cells was dissolved with isopropanol 0.4% HCl. The optical density was recorded after 15 min at 550 nm using an iMark reader (Bio-Rad, Mississauga, Ontario, Canada). IC50 was defined as the half maximal inhibitory concentration required to inhibit proliferation.

### 4.3. Cell Apoptosis Assay

The impact of Rapamycin on programmed cell death was evaluated utilizing the Annexin V-Propidium Iodide Apoptosis kit (Biolegend, San Diego, CA, USA). Briefly, after 24 h of Rapamycin treatment, Ca9-22 cells were harvested, washed and then resuspended in 100 µL Annexin V binding buffer supplemented with 5 μL Annexin V and 5 μL PI. Following a 20 min incubation at ambient temperature, 400 μL of Annexin V binding buffer was introduced and flow cytometry analysis was carried out employing either the BD LSR II or the BD FACSCanto II cytometers featuring the FACSDiva software v. 6.1.3. These experiments were carried out four times. The total events were classified as viable (AnxV/PI-), early apoptotic (AnxV+/PI-), late apoptotic (AnxV+/PI+) and necrotic cells (AnxV-/PI+).

### 4.4. Cell Autophagy Assay

The potential effect of Rapamycin on Ca9-22 cell autophagy was evaluated by flow cytometry, as previously described [14,74]. Briefly, following 24 h exposure to 10 µM of the drug, the Ca9-22 cells were collected and resuspended in 500 μL culture medium containing 1:5 red autophagy probe (Immunochemistry Technologies, Davis, CA, USA) and then incubated in darkness for 1 h at 37 °C. Following two washes with PBS 1X, 500 μL 1X assay buffer were added prior to flow cytometry analysis by the BD LSR II or BD FACS Canto II system (BD Bioscience, Mississauga, ON, Canada) equipped with FACS Diva Software v. 6.1.3.

### 4.5. RNA Extraction and Reverse Transcription

In summary, Ca9-22 cells were exposed to 10 μM Rapamycin for a duration of 24 h. Total RNA was isolated following the manufacturer’s guidelines using the RNeasy mini kit (Qiagen, Toronto, ON, Canada). RNA concentration and purity were evaluated using a NanoDrop spectrophotometer (Thermo Fisher, Waltham, MA USA). The RNA was subsequently reverse transcribed into cDNAs using the RT^2^ First Strand Kit (Qiagen, 330404).

### 4.6. Gene Expression Using RT^2^ Profiler PCR Array

To investigate the genetic variations related to Rapamycin exposure, we used the RT^2^ Profiler PCR arrays corresponding to cell cycle (Qiagen, PAHS-020ZD), apoptosis (Qiagen, PAHS-012ZD) and autophagy (Qiagen, PAHS-084ZF). These arrays allowed assessment of the expression of a set of 84 genes associated with the tumorigenesis processes. Briefly, the PCR mixture was prepared, which included 1350 μL of SYBR Green Mastermix (Biorad, 64204590), 102 μL of cDNA and 1248 μL of RNase-free water. Afterwards, 25 μL of this mixture was added to each well of the RT^2^ profiler plate. After a brief centrifugation step for 1 min at 1000× *g* at 25 °C, real-time PCR was conducted. The data acquired by real-time PCR were analyzed using the 2^−ΔΔCT^ method to determine relative gene expression and the fold changes between cells not exposed to and treated with Rapamycin. Only at least two-fold variations relative to the controls were taken into consideration. CT values were then extracted and compiled into a table, which was subsequently uploaded to the online platform for analyzing data, accessible at http://www.qiagen.com/geneglobe. Gene expression was normalized based on an automated selection from a comprehensive set of reference genes. Control samples were untreated cells; however, test groups were cells treated with 10 µM of Rapamycin corresponding to IC_50_.

### 4.7. Gene–Gene Interaction Networks

The Gene MANIA website was employed to predict genes with similar functions and to construct a network of gene–gene interactions (GGIs). Genes with similar characteristics were situated in the outer circle, whereas hub genes occupied the inner circle. The node color corresponded to the gene function and the line color indicated the type of genetic interaction.

### 4.8. Statistical Analysis

The GraphPad Prism 7 software was used to compare between experimental (treated) and control (untreated) groups. Significant differences were assessed by means of Student’s t-test and ANOVA tests. The error bars depict the mean value ± SEM. Significant data corresponded to *p*-values less than 0.05 (* *p* < 0.05, ** *p* < 0.01, *** *p* < 0.001 and **** *p* < 0.0001).

## 5. Conclusions

Rapamycin’s anticancer action by inhibiting the cell cycle and inducing apoptosis and autophagy through inhibiting certain associated genes may be a serious therapeutic target to exploit for the treatment of oral cancer. The study of the effect of Rapamycin on cell cycle/apoptotic and autophagic methylation profile genes would be a perspective for understanding how Rapamycin inhibits the expression of these genes.

## Data Availability

All data generated in the current study are included in this published article.
